# Determinants of quality of life following resection of skull base tumors: a systematic review

**DOI:** 10.3389/fonc.2024.1473261

**Published:** 2024-12-20

**Authors:** Veronika Sperl, Thomas Rhomberg, Thomas Kretschmer

**Affiliations:** Department of Neurosurgery and Neurorestoration, Klinikum Klagenfurt am Wörthersee, Klagenfurt, Austria

**Keywords:** quality of life, skull base surgery, neurooncology, systematic review, patient-reported outcome measures

## Abstract

**Background:**

Skull base tumors represent a small subset of intracranial neoplasm. Due to their proximity to critical neurovascular structures, their resection often leads to morbidity. As a result, surgical interventions can exacerbate symptoms or cause new deficits, thereby impacting the patients’ perceived quality of life (QoL). The factors influencing QoL in patients with skull base tumors remain underexplored. This systematic review aims to synthesize current research on QoL outcomes and identify potential factors influencing QoL in these patients.

**Methods:**

A systematic literature review was conducted in PubMed using the keywords “Skull Base” AND “Quality of Life.” A total of 815 studies published up to January 31, 2024, were screened. After abstract review, 656 studies were excluded, and 159 studies underwent full-text review. The wide variability in study methodologies and utilized QoL instruments made only a descriptive comparison possible.

**Results:**

In total, 113 studies were systematically reviewed. Publications focusing on the same tumor type or localization were compared. The majority of studies addressed tumors of the anterior skull base, with pituitary adenomas, meningiomas and vestibular schwannomas being the most commonly represented. The impact of surgery on QoL is often underestimated by caregivers and has a more profound effect on patients than expected by surgeons. A transient decline in QoL after surgery was observed across almost all studies regardless of localization and entity. Factors influencing QoL included age, gender, tumor localization, surgical approach, tumor type, extent of resection, preoperative clinical status and neurological deficits. Radiotherapy and recurrent surgeries were predictors of poorer QoL. Early psychological intervention in complex tumors appears to enhance QoL. Some successful sealing techniques, such as nasoseptal flaps and lumbar drains, affected QoL. However, variability in study methodologies reduced the validity of the findings.

**Conclusion:**

This review highlights the significant impact of skull base tumor surgery on patients’ QoL. Given the major oncological and surgical challenges presented by skull base tumors, their treatment significantly affects QoL, and gross total resection (GTR) should not always be the primary goal. Additionally, recognizing and addressing the modifiable and non-modifiable factors influencing QoL is crucial for improving patient outcomes and providing personalized care.

## Introduction

Tumors at the skull base, while representing only a small subset of intracranial neoplasms, present considerable challenges in neurosurgery due to their proximity to critical neurovascular structures. This anatomical complexity necessitates highly specialized surgical approaches, often carrying a significant risk of morbidity (1).

Skull base tumors are a diverse group of adult and pediatric neoplasms and exhibit considerable heterogeneity in their originating tissue and dignity, encompassing a wide range of different histological tumor entities (2). These tumors typically arise outside the brain parenchyma and can develop in distinct anatomical compartments of the skull base such as the meninges (e.g. meningiomas), sellar region (e.g. pituitary adenomas or craniopharyngiomas), cranial nerves (e.g. schwannomas) or bone and cartilage tissue (e.g. chordomas or chondrosarcomas) (3). The estimated incidence of these tumors varies significantly depending on the tumor type, with pituitary adenomas being the most common, occurring at an incidence of approximately 2.7 per 100,000 individuals in the United States (4).

Most skull base tumors show limited responsiveness to chemotherapy. As a result, surgical resection and radiotherapy remain the primary therapeutic modalities (2). However, the proximity of these tumors to critical neurovascular structures, such as the cranial nerves, the brainstem and major blood vessels, poses a significant risk during surgical intervention, often making complete resection difficult or impossible (1). Consequently, surgery is typically the initial step in treatment, aimed at reducing tumor burden, followed by adjuvant radiotherapy to control residual tumor tissue.

Despite the benefits of surgery and radiotherapy, certain tumor types, such as sarcomas and chordomas, demonstrate resistance to conventional radiation therapy. In these cases, more advanced therapeutic techniques, such as particle beam therapy, have emerged as promising additional tools, offering enhanced precision and efficacy in targeting radioresistant tumors while sparing surrounding healthy tissue (5).

Historically, research on skull base tumors has concentrated on clinical endpoints such as mortality rates, surgical complications, the extent of tumor resection, responses to radiation therapy and overall survival rates (6–8). These factors are crucial for evaluating the efficacy of treatment modalities and for predicting long-term outcomes. However, they do not fully capture the comprehensive impact of the disease and its treatment on patients’ daily lives.

Quality of life (QoL) has emerged as an equally important outcome measure. It is a multidimensional construct that encompasses physical, psychological and social aspects of health from the patient’s perspective (9). These dimensions help understand the broader impacts of medical interventions, extending beyond immediate clinical outcomes. The diagnosis of a skull base tumor itself can carry a significant psychological burden, potentially leading to anxiety and depression (10, 11). Surgical interventions, while often necessary for managing or curing the disease, can exacerbate these issues, especially if they result in noticeable physical or functional deficits.

The recovery period for these patients can be demanding, involving rehabilitation, adjustment to new limitations, undergoing adjuvant therapy and coping with the fear of recurrence, all of which can further influence the patient’s quality of life (12, 13).

In the last decades, there were no validated instruments available specifically designed to measure such complex outcomes. As a result, tools like custom questionnaires and the Karnofsky Performance Status Scale (KPS) were employed to indirectly assess QoL. Originally developed to evaluate the ability of cancer patients to perform ordinary tasks, the KPS primarily quantifies a patient’s functional status and predicts their capacity to endure therapies. This scale is used predominantly by physicians to measure physical independence, rather than capturing the subjective well-being of the patient (14).

Over time, more advanced QoL assessment tools have been developed that directly measure the patient’s experience, such as the 36-Item Short Form Survey (SF-36). The SF-36 is a reliable and validated instrument which consists of 36 questions split into eight categories that explore both the physical and psychological dimensions of health, including physical functioning, role limitations due to physical or emotional problems, vitality, emotional well-being, social functioning, pain and general health perception (15). This multifaceted approach to assess various health dimensions makes the SF-36 a widely used questionnaire across various fields of medicine, not just skull base oncology.

While general QoL instruments like the SF-36 cover a broad array of health aspects, certain anatomical locations require more specialized instruments. The Anterior Skull Base Questionnaire (ASBQ), for instance, is specifically designed to assess QoL facets relevant to anterior skull base pathologies. It provides a validated and comprehensive evaluation through 35 questions divided into six subdomains: performance, physical function, energy and vitality, pain, specific symptoms and emotional impact (16).

Other QoL instruments frequently utilized in skull base surgery, such as the Anterior Skull Base Nasal Inventory (ASK-12) and Sinonasal Outcome Test (SNOT-22), focus on sinonasal quality of life. These tools primarily assess nasal symptoms, neurological symptoms, emotional burden and quality of sleep, thus addressing only specific components of the overall QoL (17, 18).

While a wide variety of validated QoL instruments are available today, the ones mentioned above are the most frequently used to assess QoL in the studies we have reviewed.

This systematic review aims to investigate and mine current research focusing on QoL outcomes following the resection of skull base tumors. We will examine how these outcomes are assessed, the tools used to measure QoL, and the effect of various surgical approaches on patient-reported quality of life. By highlighting patient-centered measures, we aim to promote a more comprehensive understanding of treatment impacts, guiding both clinical decision-making and patient care strategies in skull base oncology.

## Methods

**Figure 1:**
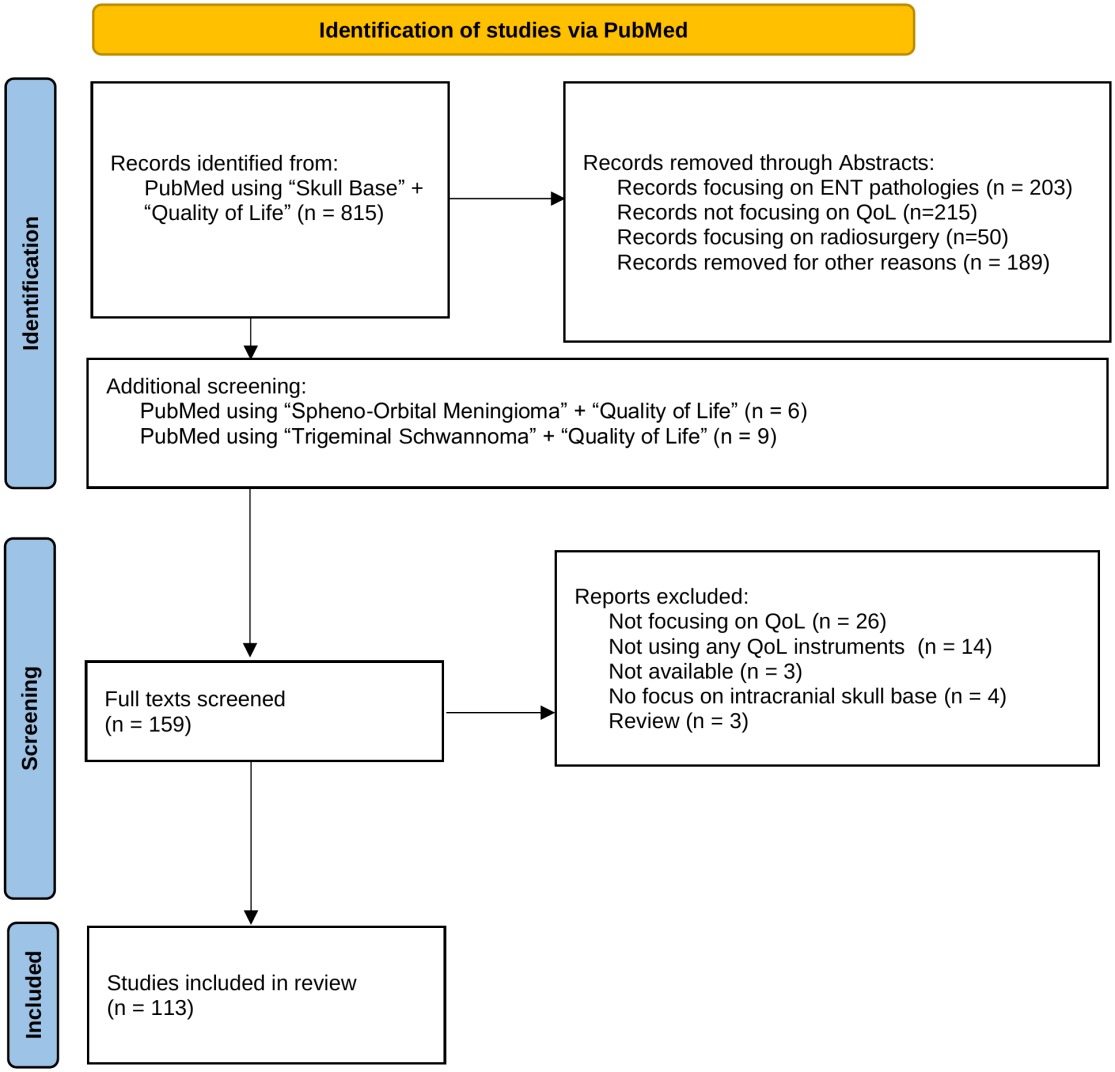
This flow chart outlines the systematic process of selecting studies for inclusion in the review, detailing the number of records identified, screened, and assessed for eligibility, as well as the reasons for exclusions at each stage.

To ensure a robust and transparent approach to our literature search and analysis, this systematic review is designed to comply with the PRISMA guidelines (19), as illustrated by the PRISMA flowchart (**Figure 1**).

We conducted the systematic literature review by searching PubMed using the keywords “Skull Base” AND “Quality of Life.” This search included all studies published up to January 31, 2024. Our initial search yielded 815 publications. Following a screening of abstracts, 159 studies were selected for detailed evaluation. We excluded 656 studies based on the following criteria: lack of focus on quality of life, primary involvement with ENT pathologies, studies evaluating radiosurgery techniques, or those not centrally addressing skull base pathologies.

The selected 159 articles underwent full-text review by the first two authors. Further exclusions were applied for studies that did not employ a validated quality of life assessment tool.

In cases where certain tumor types were underrepresented, we performed additional, targeted literature searches and cross-referenced existing findings. This methodological step was crucial to ensure that no significant studies were overlooked, resulting in the inclusion of one more study.

The final collection comprised 113 studies and we systematically compared the outcomes across these studies to identify factors that significantly impact the quality of life following skull base tumor resection. Publications focusing on more than one tumor identity were discussed for every single tumor identity. In the corresponding tables, these studies have been marked with an asterisk (*). Additionally, our analysis assessed the variety and frequency of quality of life assessment tools used, and examined the distribution of studies by tumor type and location to identify any patterns or gaps in the research landscape. To determine the country of origin for each study, we recorded the country of the first author’s affiliated institution.

Figures presented in this study were created using Microsoft PowerPoint for initial layouts and basic graphics and refined in Affinity Designer 2.5. The ggplot2 library in R was used for the visualization of bar charts.

## Results

**Figure 2:**
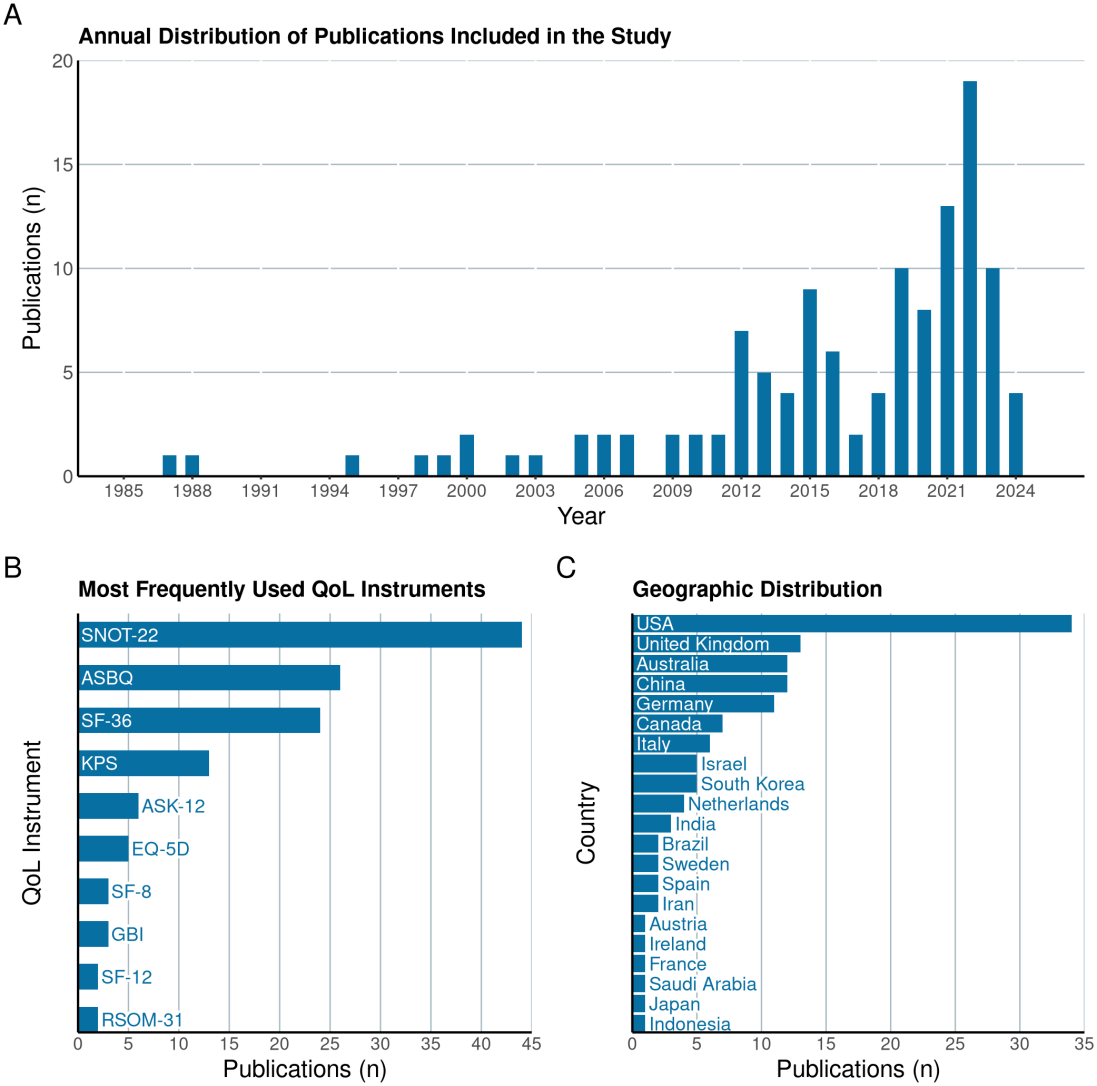
Illustration of the annual distribution of publications **(A)** included in the systematic review, highlighting trends in research volume over time. The figure also details the most frequently utilized Quality of Life (QoL) instruments in these studies **(B)** and the countries of origin of the included research **(C)**.

**Figure 3:**
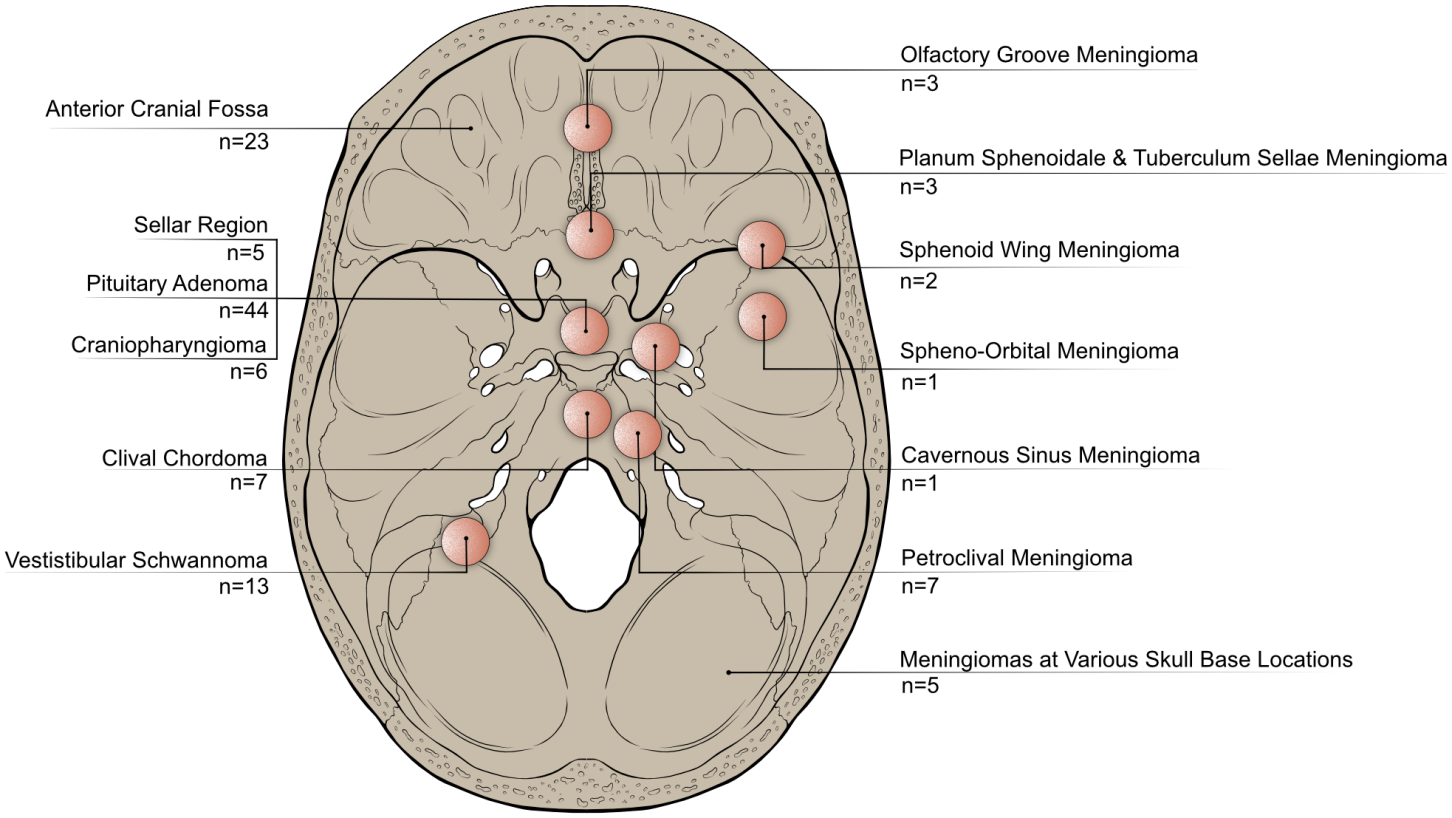
Categorization of publications included in this review based on the anatomical locations or tumor entities they focus on. This highlights variations in research focus across different anatomical regions or types of skull base tumors.

113 articles were included in this review, with the majority being published after 2010 (**Figure 2A**). The five most commonly utilized quality of life assessment tools included the SNOT-22 (n=44), the ASBQ (n=26), the SF-36 (n=24), the KPS (n=13) and the ASK-12 (n=6) (**Figure 2B**). The majority of the studies originated from the USA (n=34), United Kingdom (n=13), Australia (n=12), China (n=12), and Germany (n=11) (**Figure 2C**). Each study included in this review specifically targeted distinct tumor types or particular regions of the skull base (**Figure 3**).

Most publications focused on pituitary adenomas (n=44), different tumor identities located in the anterior skull base (n=23) and meningiomas (n=22).

### Tumors of the anterior skull base

Tumors of the anterior skull base constitute a significant portion of skull base tumors, spanning a wide spectrum of different benign and malignant lesions. Historically, open surgical approaches were standard in the treatment of these lesions, including those that are highly invasive and often require extensive surgical intervention. Studies identified that focus on these tumors have been summarized in **Table 1**.

**Table 1:** Studies investigating QoL in patients after resection of various tumors located in the anterior skull base.

First Author	Time of Surgery	Patients (n)	Surgical Approach	Follow-Up (months)	QoL Instruments	Factors Influencing QoL
(20)	1993-1997	18	Transbasal	Up to 60	SF-36	No significant factor.
(21)	2017-2018	46	Endoscopic endonasal	11,8 (mean)	SNOT-22	Temporary QoL impairments after surgery.
(22)	2010-2019	727	Various endoscopic endonasal approaches	Up to 24	SNOT-22	Mometasone irrigation after surgery improved sinonasal QoL.
(23)	Not specified	27	Various microsurgical approaches	At least 6	CES-D, ALHR, MDS	Recurrence, radiotherapy and MDS related to lower QoL.
(24)	2002-2007	48	Subcranial	28 (median)	ASBQ	Worse QoL in patients with malignant histopathology and adjuvant radiotherapy.
(25)	2008-2010	41	Expanded endonasal approach	At least 12	ASBQ	Female gender associated with poorer postsurgical QoL.
(26)	2014-2017	51	Various endoscopic endonasal approaches	At least 3	ASBQ, SBI, SNOT-22	Female gender, recurrent surgery and radiotherapy linked to poorer QoL.
(27)	2010-2013	250	Endoscopic endonasal	6	SNOT-22	Reconstruction with calcium hydroxyapatite and postoperative mucosal edema negatively impacted sinonasal QoL.
(28)	2010-2020	96	Endoscopic endonasal	6	SNOT-22	Short-term sleep impairment after surgery.
(29)	2014-2018	87	Endoscopic endonasal	6	UPSIT	Omega-3 supplementation linked to protective postoperative olfactory function.
(30)	2008-2010	36	Endoscopic endonasal	3	SNOT-20	Sinonasal QoL unaffected by surgery.
(31)	2009-2010	11	Endoscopic endonasal	> 5	SNOT-22, SF-12, HUI-2	QoL unaffected by surgery.
(32)	2012-2016	148	Endoscopic endonasal	>5	SNOT-22	Temporary QoL impairments after surgery.
(33)	2003-2010	78	Subcranial and endoscopic endonasal	Up to 12	ASBQ	Lower QoL in females in endoscopic group and adjuvant radiation therapy worsens QoL.
(34)	Not specified	38	Expanded endoscopic endonasal	60	ASBQ	Surgery-related lumbar drain insertion increases complications and reduces QoL.
(35)	1996-2004	19	Subcranial	44 (mean)	EORTC QLQ-30, EORTC QLQ-H&N35	Reduced QoL after surgery with no significant factors identified.
(36)	1995-2001	14	Not specified	40 (mean)	QoLI	Reduced QoL after surgery with no significant factors identified.
(37)	1994-2002	69	Subcranial	Up to 6	Custom Questionnaire	Old age, malignancy, comorbidity, radiotherapy and extensive surgery identified as negative QoL prognostic factors.
(38)	1992-2003	18	Various open and endoscopic approaches	30 (mean)	UoW QoL questionnaire, HADS	One-third of skull base malignancy patients exhibited significant mental distress and psychiatric morbidity.
(39)	2021-2021	40	Endoscopic endonasal	Up to 24	ASBQ, SNOT-22	Temporary declines in olfactory, vision and taste function may lead to decreased short-term QoL.
(40)	1997-2010	153	Endoscopic endonasal	Up to 12	ASBQ	Age, expanded surgical approach and postoperative radiotherapy linked to worse QoL.
(41)	2005-2015	26	Anterolateral craniofacial resection with orbital exenteration	Up to 24	SF-8, HADS	80% of patients needed psychiatric intervention.
(11)*	2013-2017	23	Transnasal and transcranial	12	SF-36, EQ-5D various depression and anxiety scores	QoL improvement and psychological relief after surgery.
(42)	2007-2019	57	Endoscopic endonasal	Not specified	ASBQ	QoL improvement at 1 month postoperatively, with continued improvement stabilizing at 6 months after surgery.
(43)	2016-2022	50	Endoscopic endonasal	12	SNOT-22, ASBQ	Loss of olfaction reduces QoL, while visual improvement enhances QoL.

Publications focusing on more than one tumor identity have been marked with an asterisk (*). These publications were discussed for every single tumor identity.

Recent advancements have increasingly supported the use of endoscopic endonasal approaches for treating anterior skull base lesions, where appropriate. While these techniques are not suitable for all tumors, they have been shown to improve QoL outcomes when compared to traditional open approaches like the subcranial approach, particularly as measured by the ASBQ (24). Furthermore, long-term QoL studies affirm the benefits of endoscopic methods for eligible lesions at the anterior skull base (26).

Earlier studies highlight the challenges associated with open surgery. High morbidity rates and significant disruptions in returning to work were noted among patients undergoing complex tumor resections (20). These issues are reflected in the diminished role function scores, indicating a negative impact on the patients perceived capacity to work (35, 36).

Studies suggests that QoL typically declines immediately following anterior skull base tumor resection, but generally returns to baseline within 6 to 12 months after surgery (24, 28, 37). Emotional and financial difficulties, as well as sleep disturbances, are common after surgery (35). Additionally, sinonasal QoL issues, such as nasal crusting or olfactory impairments, affect approximately two-thirds of patients (23). These conditions, as measured by the SNOT-22, often show improvement as early as 3 to 6 months following surgery (21, 27, 32, 39).

Some studies focusing specifically on meningiomas in the anterior skull base demonstrated significant improvement in QoL as early as one month after resection, with further improvements observed up to the six-month follow-up (42). However, more aggressive resections (Simpson Grade I) tend to result in higher rates of cranial nerve deficits (44). While visual improvement after surgery significantly impacts QoL, the loss of olfaction or taste is considered less critical (45). These neurological deficits were found to significantly decrease QoL (23, 39).

Significant disparities in QoL outcomes have been observed among patients with malignant and benign skull base pathologies (24). Patients with malignant pathologies experienced significantly lower QoL scores six months after surgery. However, there was a notable improvement in their QoL twelve months after surgery, as measured by the SNOT-22, HUI-2, and SF-36 (24, 31). In contrast, QoL scores for patients with benign tumors remained stable throughout the postoperative period (24).

Patients with malignant tumors of the anterior skull base often experience significant mental distress and psychiatric morbidity, necessitating the use of psychotropic medication in up to 80% of cases (35, 38, 41). Those undergoing extensive open cranial surgery may benefit from early psychiatric and psychological interventions, which can help them return to normal psychological health approximately two years post-surgery (41). In contrast, patients with benign lesions often experience significant psychological relief following tumor resection, whether through open or endoscopic approaches (46).

Adjuvant radiotherapy significantly worsened physical functioning, role performance and vitality. Along with recurrent surgery, it was strongly linked to poorer quality of life outcomes, measured using the ASBQ, SBI, and SNOT-22 test (24, 26, 36, 47).

Several studies identified female gender as a predictor of poorer QoL outcomes following surgery, with significant reductions in all domains of the ASBQ. Female patients reported decreases in general performance, physical function, vitality, pain and emotional impact by 18 to 32%, whereas male patients noted improvements of up to 18% in these areas (24–26).

Other factors linked to poorer postoperative QoL include older age, comorbidities and more extensive surgeries (37). The use of a preventive lumbar drain for cerebrospinal fluid (CSF) leaks in transsphenoidal endoscopic tumor resection was associated with increased complications, longer hospital stays and overall decreased QoL (34).

Conversely, certain postoperative regimes, such as omega-3 supplementation after endoscopic transnasal surgery, might improve QoL due to its potential protective effects on olfactory function (29). Postoperative irrigation with mometasone twice a day significantly reduced postoperative SNOT-22 scores compared to budesonide and saline (22).

### Tumors of the sellar region

The sellar region is the site of origin for various tumors arising from different tissue types, with adenomas and meningiomas being the most common. In recent years, the endoscopic transnasal approach has become a widely adopted surgical approach when suitable, leading to numerous studies that evaluate QoL using sinonasal QoL instruments such as the ASK-12 and SNOT-22 test **Table 2**.

**Table 2:** Studies investigating QoL in patients after surgery of various different tumors in the sellar region.

First Author	Time of Surgery	Patients (n)	Surgical Approach	Follow-Up (months)	QoL Instruments	Factors Influencing QoL
(48)	2016-2017	34	Endoscopic endonasal	6	SF-36, ASK-12, SNOT-22	Significant postoperative improvement in SF-36 scores.
(49)	2010-2014	46	Endoscopic endonasal	67 (mean)	SNOT-22, LMS	Younger patients experienced a higher rate of QoL deterioration.
(50)	2012-2017	767	Endoscopic endonasal	6	SNOT-20	The extended endonasal endoscopic approach resulted in worse QoL.
(51)	2014-2017	169	Endoscopic endonasal	6	SNOT-22	No difference in sinonasal QoL between baseline and 6 months after surgery.
(52)	Not specified	158	Endoscopic endonasal	12	ASBQ, SNOT-22	Reconstruction with a nasoseptal flap does not affect long-term QoL.

While many studies report no significant change in the long-term ASK-12 and SNOT-22 scores before and after tumor resection in the sellar region, the SNOT-22 scores can deteriorate following surgery in the sellar region, typically worsening for a period of 3 to 12 weeks before returning to baseline levels within 3 to 6 months (49). In one study, tumors requiring an extended endoscopic endonasal approach were associated with worsened sinonasal QoL compared to those treated with a standard transsellar approach, measured by the SNOT-22 (50). However, other studies using the same measure reported no decline in sinonasal QoL in patients undergoing the extended approach (51). In contrast, QoL assessments using the SF-36 questionnaire generally show a significant improvement after surgery (48, 51). To address CSF leaks, a common complication of transnasal surgery, nasoseptal flaps are frequently used for reconstruction. However, these flaps seem to have little effect on the long-term quality of life outcomes (52).

Age significantly influences postoperative quality of life outcomes, with younger patients exhibiting a greater deterioration in quality of life following the resection of tumors in the sellar region compared to older individuals (49).

### Pituitary adenomas

**Table 3** provides a summary of the studies identified that predominantly focus on the quality of life in patients undergoing pituitary adenoma surgery. Studies encompassing multiple tumor types, including those involving patients with pituitary adenomas, are specifically annotated in the table.

**Table 3:** Studies investigating QoL in patients after surgery of pituitary adenomas.

First Author	Time of Surgery	Patients (n)	Surgical Approach	Follow-Up (months)	QoL Instruments	Factors Influencing QoL
(53)	2018-2020	128	Endoscopic endonasal	14	ASK-12	Temporary decline in sinonasal QoL, recovered one month after surgery.
(43)	2016-20221	366	Endoscopic endonasal	Up to 12	ASBQ	Temporary decline in QoL, recovery 3 weeks after surgery with improvement above baseline afterwards.
(54)	2014-2016	101	Endoscopic endonasal	Up to 12	EES-Q	Time after intervention, male gender and older age positively influenced postoperative QoL.
(55)	Not specified	49	Endoscopic endonasal	At least 2	ENSQ6, SNOT-22	History of radiotherapy linked to impaired sinonasal QoL and sleep disturbances.
(56)	Not specified	20	Endoscopic endonasal	Up to 6	HADS, SNOT-20	Surgery had no influence on QoL.
(57)	2015-2018	62	Endoscopic endonasal	Up to 12	ASK-12, SF-12	Improvement in visual field deficits and time after intervention correlated with improved QoL after surgery.
(58)	2016-2017	60	Endoscopic endonasal	Up to 21	ASK-12	QoL unaffected by choice of endoscopic approach.
(59)	2019-2020	15	Endoscopic endonasal	Not specified	SNOT-22, Semi-structured interviews	Olfactory and breathing difficulties are major physical and psychological factors that reduce QoL.
(60)	2019-2021	58	Microscopic and endoscopic	Up to 3	SNOT-22, ASK-12, SF-36	QoL unaffected by surgical approach.
(61)	2019-2020	40	Endoscopic endonasal	Up to 6	SNOT-22, SF-36, CSS	Reduced sinus headaches with bilateral paraseptal approach.
(62)	2015-2019	109	Endoscopic endonasal	6	SNOT-22, EQ-5D	No previous sinonasal surgery associated with fewer nasal symptoms after surgery.
(63)	2016-2020	304	Endoscopic endonasal	Up to 12	ASBQ, SNOT-22	Frail patients experience the same QoL benefits from surgery as non-frail counterparts
(64)	2015-2018	42	Endoscopic endonasal	Up to 12	SF-36, SNOT-22	Improvements after surgery in physical, mental and nasal functionality as perceived by patients.
(65)	2010-2013	81	Endoscopic endonasal	16 (median)	ASBQ, SNOT-22	Total resection correlated with improved postoperative QoL.
(65)	2010-2012	40	Endoscopic endonasal	Up to 12	ASBQ, SNOT-22	Increased intranasal area after surgery had no effect on sinonasal QoL.
(66)	2014-2018	109	Endoscopic endonasal	Up to 4	SNOT-22	Nasoseptal flap usage and prior smoking may adversely impact postoperative QOL.
(47)	Not specified	82	Endoscopic endonasal	6	SNOT-22	Preserving the middle turbinate has no significant negative effects on sinonasal QoL.
(67)	Not specified	159	Endoscopic endonasal	36 (mean)	GBI	Cushing patients and those with preoperative visual impairments reported the greatest postoperative QoL improvements.
(68)	2016-2019	113	Endoscopic endonasal	3	SNOT-22, ASBQ	Postoperative prophylactic antibiotics showed no positive impact on sinonasal QoL.
(63)	2016-2020	304	Endoscopic endonasal	12	SNOT-22, ASBQ	Prolactinomas and non-functioning pituitary adenomas show QoL improvements as early as 3 months after surgery.
(69)	2016-2018	103	Endoscopic endonasal	6	SF-36	Problems with smell and taste significantly affect patient QoL.
(70)	2010-2012	85	Endoscopic endonasal	Up to 12	ASBQ	Recovery of smell, taste and visual impairments positively influenced patient QoL.
(71)	Not specified	38	Endoscopic endonasal	3	SF-36, RSOM-31	Reconstruction with a vascularized flap further decreased postoperative QoL.
(72)	2010-2011	39	Endoscopic endonasal	3	SNOT-22	Temporary decline in sinonasal QoL, recovered three months after surgery.
(73)	2014-2017	49	Endoscopic endonasal	6	SNOT-22, ASBQ	QoL improved 4 to 6 months after surgery, specifically in domains related to pain and vitality.
(74)	2013-2018	243	Endoscopic endonasal	3	SNOT-22	Early resolution of nasal crusting associated with better QoL.
(75)	Not specified	149	Endoscopic, Transnasal microscopic, sublabial	Not specified	SNOT-22, SF-36, CSS	Disease-specific QoL was superior with the endoscopic approach, resulting in reduced long-term sinonasal morbidity.
(46)*	2013-2017	17	Endoscopic endonasal	12	SF-36, EuroQoL, various anxiety and depression scales	Postoperative QoL improvement and psychological relief.
(76)	2012-2013	55	Endoscopic endonasal	3	SNOT-20, ASK-12	Endoscopic modified transseptal transsphenoidal approach showed better sinonasal QoL compared to endoscopic transnasal transsphenoidal approach.
(77)	2011-2013	100	Endoscopic endonasal	6	ASK-12, SF-8	Sinonasal QoL after endoscopic pituitary surgery hits a low at 2 weeks and recovers by 3 months after surgery.
(78)	2011-2013	218	Endoscopic and microscopic endonasal	6	ASK-12, SF-8, EQ-5D	No difference in postoperative QoL between surgical techniques.
(79)	2012-2014	81	Endoscopic endonasal	3	SNOT-22	Better sinonasal QoL 3 months after surgery in the transseptal transsphenoidal approach group.
(80)	2011-2014	106	Endoscopic endonasal	At least 12	SNOT-22	ACTH-secreting adenomas associated with poorer sinonasal QoL.
(81)*	2009-2012	5/55	Endoscopic endonasal	12	SF-36, RSOM-31, BAST-24	Skull base surgery with an expanded endonasal approach had no negative long-term impact on QoL
(82)	2007-2016	18	Endoscopic endonasal	3 (mean)	SF-36	QoL improved 3 months after surgery compared to preoperative levels.
(83)	2018-2020	46	Endoscopic endonasal	3	ASK-12	Sinonasal QoL transiently declined, while olfaction and gustation showed long-lasting declines.
(84)*	2008-2011	47/85	Endoscopic endonasal	6	SNOT-22, ASBQ	Gross total resection increased postoperative QoL.
(85)	2014-2017	12/31	Endoscopic endonasal	12	SNOT-22	The use of a nasoseptal flap does not affect sinonasal QoL.
(86)*	2010-2011	38/66	Endoscopic endonasal	6	SNOT-22, ASBQ	Better short-term QoL in patients with gross total resection.
(87)	2014-2021	61/95	Endoscopic endonasal	34 (mean)	SNOT-22, ASBQ	Only one third of patients report negative sinonasal QoL.
(88)	2016-2020	451	Endoscopic endonasal	12	ASBQ	Deficient preoperative endocrine function associated with improved postsurgical QoL.
(89)	2017-2019	31/36	Endoscopic endonasal	6	SNOT-22, UPSIT	Sinonasal QoL unaffected by surgery.
(90)	2011-2012	22	Endoscopic endonasal	Up to 3	SNOT-22	Sinonasal QoL unaffected by surgery.
(91)	2000-2010	110	Endoscopic endonasal	Up to 12	RSOM-31	Hormone-secreting adenomas have the most adverse effect on QoL.

Publications focusing on more than one tumor identity have been marked with an asterisk (*). These publications were discussed for every single tumor identity.

Preoperative QoL, as measured by the ASBQ, was notably lower in female patients, those with diabetes, visual deficits, endocrinopathy, functioning adenomas, or headaches compared to patients with incidental adenomas (54, 88, 92). Additionally, QoL measured by the SF-36 questionnaire indicated decreased QoL in six of its eight domains preoperatively in patients with pituitary adenomas (82).

After surgery, QoL typically declined transiently in the first 2-4 weeks, particularly in sinonasal health and physical functioning, before improving to above baseline levels by 6-12 weeks and continuing to improve throughout the first postoperative year (43, 53, 74, 77, 82, 84, 92). Long-term improvements in QoL were observed following endoscopic surgery (65), exceeding preoperative levels (65), even among frail patients who experienced comparable visual and endocrine outcomes to their non-frail counterparts (63).

Postoperative nasal symptoms such as nasal discharge, pain and nasal whistling as well as issues with smell and taste significantly affected physical QoL (69, 87). These symptoms, peaking in the initial days after surgery (54), led to QoL impairments in domains such as sleep, mood, appetite, sexual desire, nutrition, health, hobbies and social interactions (59). However, these impairments typically resolved or significantly improved within three months after surgery, particularly in the domains of physical well-being, vitality and pain (11, 54, 57, 67, 73, 87). Several studies reported that olfactory and taste-specific QoL impairments, initially present after surgery, were no longer measurable 1 to 12 months later (53, 60, 70, 83, 89). Improvements in vision or visual field deficits were particularly associated with favorable QoL outcomes, which were measurable as early as three months after surgery (57, 67, 70).

In contrast to physical and social QoL, psychological QoL tended to improve directly postoperatively and three months after surgery, psychological QoL returned to baseline (54), with some studies reporting normalization of mental functions only after one year (57). Significant improvements in overall postoperative QoL were driven by improved emotional states of the patients (11, 73).

Previous sinonasal surgery, smoking, and the use of a nasoseptal flap were linked to worse rhinologic symptoms and QoL (62, 66, 71). Although the nasoseptal flap could cause worse sinonasal morbidity and headache in the immediate postoperative period, it did not have a long-term negative impact on QoL, with patients typically returning to baseline by 3-6 months after surgery (66, 80, 84, 91). In contrast, other studies found no impairment in sinonasal QoL and olfactory function after surgery (93, 94), even when using a nasoseptal flap (85).

Several studies demonstrated that gross total resection (GTR) resulted in better postoperative QoL compared to subtotal resection, as measured by ASBQ and SNOT-22 (65, 84, 86). However, other studies showed no significant difference in QoL based on the extent of resection (73, 74). Female sex and older age were associated with worse postoperative QoL (43, 77), although age was not a consistent factor across all studies (92).

Functioning pituitary adenomas were associated with worse QoL, as measured by RSOM-31 and EES-Q QoL instruments (54, 91), although this was not universally observed across all studies (71, 73) and some authors report a preoperative endocrinopathy as a factor associated with better postoperative QoL measured by the ASBQ-35 (92). Patients with Cushing’s disease reported significant QoL benefits from surgery, particularly in physical health domains. Prolactinoma and non-functioning pituitary adenoma patients also experienced significant QoL improvements three months after surgery (43). In contrast, ACTH-secreting adenomas were associated with worse sinonasal QoL after surgery. Tumor size did not significantly affect postoperative QoL (92).

Comparative studies of surgical approaches found that endoscopic techniques yielded better QoL outcomes measured by SF-36 and SNOT-22 compared to microscopic approaches (75). Conversely, other studies showed opposite results using the ASK, SF-8, and EQ-5D questionnaires (76, 78). Various endoscopic approaches have been explored in the literature, revealing only minor differences in QoL due to headache or olfactory function that were negligible in long-term follow-ups (47, 58, 60, 61, 79, 81, 90). Cerebrospinal fluid leaks during surgery did not significantly reduce QoL after surgery (73), although some studies noted slight negative associations (88).

### Craniopharyngioma

**Table 4** summarizes studies related to craniopharyngiomas, which frequently present surgical challenges due to their location and expansive growth. Studies involving multiple tumor types, including craniopharyngiomas, have been specifically annotated in the table.

**Table 4:** Studies investigating QoL in patients after craniopharyngioma surgery.

First Author	Time of Surgery	Patients (n)	Surgical Approach	Follow-Up (months)	QoL Instruments	Factors Influencing QoL
(95)	1996-2002	19	Various microsurgical approaches	Up to 280	SF-36, KPS	Overall high long-term QoL after surgery, with no associated factors.
(96)	2004-2013	31	Endoscopic endonasal	Up to 101	SNOT-22, ASBQ	Overall, postoperative QoL maintained at preoperative levels. Better QoL observed in patients with GTR and radiation therapy, while worse QoL was noted in patients with visual or endocrine deficits.
(97)	2001-2018	30	Transcranial and endoscopic endonasal	136 (mean)	SNOT-22, ASBQ	No difference in postoperative QoL between endonasal and transcranial approaches.
(81)*	2009-2012	3/55	Expanded endoscopic endonasal	12	SF-36, RSOM-31, BAST-24	Skull base surgery with an expanded endonasal approach had no negative long-term impact on QoL.
(86)*	2008-2011	4/85	Endoscopic endonasal	6	SNOT-22, ASBQ	Elapsed time after intervention and gross total resection increased QoL.
(84)*	2010-2011	2/66	Endoscopic endonasal	6	SNOT-22, ASBQ	Better short-term QoL in patients with gross total resection.

Publications focusing on more than one tumor identity have been marked with an asterisk (*). These publications were discussed for every single tumor identity.

A longitudinal study spanning over 20 years demonstrated that the overall QoL for patients, after resection of a craniopharyngioma, was relatively high, as measured by the SF-36 and KPS indices (95). Gross total resection is associated with a higher QoL (84, 96), while tumor recurrence or the need for additional resections tends to worsen QoL. Patients who experience visual improvement after surgery tend to report higher QoL scores, whereas persistent visual deficits lasting over a year, as well as hypopituitarism, have been shown to significantly worsen QoL (96).

Gender differences also appear to influence QoL outcomes, with female patients exhibiting lower QoL (96).

The studies we investigated found no significant differences in QoL outcomes among the various surgical techniques used for the resection of craniopharyngiomas. The primary methods fall into two main categories: endoscopic endonasal approaches and transcranial approaches (81, 96, 97). Typically, the endoscopic endonasal approach may lead to short-term, self-limited impairments in sinonasal related QoL. Moreover, techniques such as the use of a nasoseptal flap or gasket seal reconstruction in an endoscopic approach do not result in a long-term decrease in sinonasal QoL (86).

### Meningiomas

Meningiomas are among the most common types of skull base tumors and can develop in any part of the skull base, affecting various neurovascular structures and causing a wide range of symptoms. The choice of surgical approach for removing these tumors depends on their size and location, factors that can significantly influence patient QoL **Table 5**.

**Table 5:** Studies investigating QoL in patients after skull base meningioma surgery.

First Author	Time of Surgery	Patients (n)	Surgical Approach	Follow-Up (months)	QoL Instruments	Factors Influencing QoL
(98)	2004-2015	56	Transcranial and endoscopic endonasal	Up to 106	SNOT-22, ASSBQ	QoL decreased postoperatively in patients aged over 55.
(99)	2009-2011	58	Not specified	58	EORTC QLQ-C30, HADS	The majority of patients showed stable or improved QoL after surgery, with only a minority deteriorating.
(100)	2012-2016	52	Predominantly frontotemporal approach	9 (mean)	EQ-5D	Better QoL linked to female sex, no proptosis, non-frontotemporal approaches, no optic nerve compression and no surgical complications.
(101)	2016-2020	165	Transcranial	Up to 60	KPS	Longer ICU stays and hemorrhagic complications result in worse functional outcomes.
(13)	2016-2019	89	Not specified	Up to 108	SF-36, EORTC QLQ-BN20	Surgical resection of posterior fossa meningiomas resulted in lower QoL.

When the specific location of the meningioma at the skull base is not considered, resection commonly results in a temporary decline in QoL postoperatively. Typically, QoL returns to baseline levels about 12 months after surgery (99). Most studies report no significant long-term impairments in QoL following meningioma surgery (13, 99, 100). However, one study noted a decrease in QoL among patients over the age of 55 (98).

Surgical complications, including CSF leaks, wound infections and accidental cranial nerve injuries, can impact patients QoL following surgery (100). Conversely, other data indicates that surgical complications do not affect QoL (13). Severe complications such as postoperative hemorrhage and associated prolonged ICU stays can lead to functional deterioration after meningioma resection (101). Additionally, while one study observed improvements in neuropsychological functions after surgery (99), another reported no changes (13). However, neither study found these neuropsychological outcomes to influence the overall perceived QoL.

The anatomical location of meningiomas within the skull base plays a significant factor in postoperative QoL. Meningiomas situated in the posterior fossa are associated with poorer QoL outcomes compared to those located in the anterior or middle cranial fossa (13). This disparity may be attributed to the fact that the posterior fossa contains surgically highly demanding meningiomas, such as petroclival meningiomas, which present more complex challenges during resection.

### Petroclival meningiomas

Petroclival meningiomas, despite their typically benign pathology, present significant surgical challenges due to their proximity to critical anatomical structures. The complex anatomy and difficult access of this region have driven the development of surgical techniques aimed at minimizing morbidity while achieving complete resection and maintaining the QoL for patients. However, the impact of surgery on QoL is often underestimated by caregivers and has a more profound effect on patients than expected by surgeons (102). The results of our findings are summarized in **Table 6**.

**Table 6:** Studies investigating QoL in patients after petroclival meningioma surgery.

First Author	Time of Surgery	Patients (n)	Surgical Approach	Follow-Up (months)	QoL Instruments	Factors Influencing QoL
(102)	1992-1997	17	Transpetrosal	At least 12	SF-36	Postsurgical decrease in QoL. Majority with new or worsened neurological deficits.
(103)	1992-1999	19	Transpetrosal	Up to 12	SF-36, GOS	Postsurgical decrease in QoL. Majority with new or worsened neurological deficits.
(104)	1991-2004	150	Mixed; majority transpetrosal	102 (mean)	KPS	KPS decreased post-surgery, recovered after one year, and improved at long-term follow-up.
(105)	2008-2018	32	Mixed; majority retrosigmoid	35 (mean)	KPS, SF-36, GOS	GTR associated with worse postoperative QoL
(106)	1988-2012	64	Mixed; majority posterior petrosal	72 (mean)	KPS	Significant brainstem compression associated with better postoperative KPS.
(107)	1991-2010	71	Mixed; majority retrosigmoid	61 (mean)	KPS	QoL significantly correlated with extent of resection, preoperative brainstem edema, tumor-neurovascular relationships, and invasion depth into cavernous sinus.
(108)	2000-2020	25/60	Not specified	66 (mean)	Survey Battery	High overall postoperative QoL.

Postoperatively, patients typically experience a decline in QoL, which generally improves to preoperative levels within a year after surgery. Long-term follow-ups have shown that QoL even surpass preoperative levels, as measured by the KPS. However, it is important to note that severely disabled patients with a preoperative KPS score below 70 tend to have poorer outcomes one year after surgery (104).

Achieving a surgical cure often necessitates a gross total resection. However, studies have indicated that gross total resection of petroclival meningiomas can result in worse postoperative QoL compared to subtotal resection (105, 107). While aiming for gross total resection, careful attention must be paid to protecting anatomical structures, as lower cranial nerve palsies can prevent patients from returning to a normal life and significantly diminishing postoperative QoL (103). This is particularly crucial given the high risk of new postsurgical neurological deficits associated with petroclival meningioma surgery (108, 109).

Additionally, patients with preoperative brainstem compression due to the tumor have been shown to experience significantly better QoL after surgery (102, 107). The impact of other anatomical factors, such as cavernous sinus infiltration, remains controversial, with some studies indicating no effect on QoL (102) and others suggesting an influence (107).

### Sphenoid wing meningiomas

Sphenoid wing meningiomas can present a significant challenge for neurosurgeons aiming for complete and safe removal, particularly medial sphenoid wing meningiomas, which are associated with the poorest neurological functional outcomes, second only to petroclival meningiomas. These tumors negatively impact postoperative quality of life and have the highest recurrence rates among meningiomas (110–112). Two studies have investigated the quality of life in patients with sphenoid wing meningiomas, both specifically focusing on medial sphenoid wing meningiomas (**Table 7**).

**Table 7:** Studies investigating QoL in patients after sphenoid wing meningioma surgery.

First Author	Time of Surgery	Patients (n)	Surgical Approach	Follow-Up (months)	QoL Instruments	Factors Influencing QoL
(113)	1985-1999	127	Orbito-zygomatic frontotemporal, pterional and subfrontal approach	82 (mean)	KPS	Large tumors linked to poorer short-term outcomes, including visual improvement and KPS score. Long-term outcomes not correlated with tumor size.
(114)	2008-2021	36	Not specified	75 (mean)	KPS	Visual impairment found as the most significant factor reducing QoL

Visual impairment has been identified as a significant factor contributing to both preoperative and postoperative reduced QoL in patients with medial sphenoid wing meningiomas that infiltrate the cavernous sinus (114).

Tumor recurrence and progression pose the major long-term risks following resection and the initial surgery is of crucial importance. It was observed that larger medial sphenoid wing meningiomas are associated with poorer immediate clinical outcomes, including less visual improvement and lower KPS scores and present greater challenges for complete removal. However, in the long-term, tumor size did not correlate with overall outcomes measured by KPS (113).

### Spheno-orbital meningiomas

Spheno-orbital meningiomas are rare and our search identified only one study (**Table 8**) examining the QoL following their resection. This study reported a significant improvement in QoL, as measured by the European Organization for Research and Treatment of Cancer Quality of Life Core Questionnaire (EORTC QLQ). However, the analysis was limited to comparing preoperative QoL with assessments made three months after surgery and they identified no factors that significantly influenced the QoL outcomes (115).

**Table 8:** Studies investigating QoL in patients after spheno-orbital meningioma surgery and cavernous sinus meningioma.

First Author	Time of Surgery	Patients (n)	Surgical Approach	Follow-Up (months)	QoL Instruments	Factors Influencing QoL
(115)	2016	40	Not specified	3	EORTC QLQ-C30	Postoperative significant improvement in QoL across all subcategories after spheno-orbital meningioma resection.
(116)	1996-2014	65	Mixed; Majority frontotemporal orbitozygomatic	Up to 199	KPS	Patients undergoing adjuvant stereotactic radiosurgery after cavernous sinus meningioma resection showed a tendency for improved KPS.

### Cavernous sinus meningiomas

Cavernous sinus meningiomas are the most prevalent primary tumors of the cavernous sinus, yet they comprise only about 1% of all intracranial meningiomas (117). A single study investigating the QoL of patients with cavernous sinus meningiomas was found (**Table 8**). This study indicated a tendency for improved KPS scores in patients who underwent adjuvant stereotactic radiosurgery compared to those who had only microsurgical resection, potentially due to better tumor control; however, the changes were not statistically significant (116).

### Olfactory groove meningiomas

Olfactory groove meningiomas, which develop above the cribriform plate, can grow to substantial sizes before detection (118). The resection of these tumors can be achieved through various surgical approaches, depending on the surgeon’s preference and the tumor size. We identified three studies examining the QoL in patients with olfactory groove meningioma (**Table 9**).

**Table 9:** Studies investigating QoL in patients after olfactory groove meningioma surgery.

First Author	Time of Surgery	Patients (n)	Surgical Approach	Follow-Up (months)	QoL Instruments	Factors Influencing QoL
(119)	2009-2019	4	Endoscopic Transnasal	22 (mean)	SS-12	Endoscopic endonasal approach effectively preserved smell.
(120)	2005-2023	57	Supraorbital keyhole approach and traditional transcranial approaches	39 (mean)	ASBQ	No QoL difference among surgical approaches. Keyhole approach resulted in shorter hospital stays.
(121*)	1998-2008	34/52	Superior interhemispheric approach	57 (mean)	KPS, RNLI	No significant factors found.

Publications focusing on more than one tumor identity have been marked with an asterisk (*). These publications were discussed for every single tumor identity.

In selected cases, the endoscopic transnasal approach has demonstrated a good rate of smell preservation (119), while the supraorbital keyhole approach is associated with reduced postoperative edema and shorter hospital stays compared to traditional open approaches (120). However, the choice of surgical approach did not affect the overall QoL for these patients (120). One study using the Reintegration to Normal Living Index (RNLI) found that patients undergoing resection via the superior interhemispheric approach experienced a moderately reduced QoL, without identifying any specific factors influencing this outcome (121).

### *Tuberculum sellae* and *planum sphenoidale meningiomas*

Tuberculum sellae and planum sphenoidale meningiomas originate in close proximity. Given that most studies we have reviewed involve cohorts with both types of meningiomas, we have combined them into a single section (**Table 10**). These studies primarily focus on evaluating the effectiveness of various surgical techniques and also assess quality of life outcomes.

**Table 10:** Studies investigating QoL in patients after tuberculum sellae & planum sphenoidale meningioma surgery.

First Author	Time of Surgery	Patients (n)	Surgical Approach	Follow-Up (months)	QoL Instruments	Factors Influencing QoL
(122)	2012-2021	38	Unilateral subfrontal and endoscopic endonasal	66 (mean)	KPS	KPS increased by around 15 points after surgery. No significant factors identified.
(123)	2017-2020	20	Supraorbital keyhole approach and endoscopic endonasal	12	SF-36	No QoL difference between the two groups.
(121*)	1998-2008	18/52	Superior interhemispheric approach	57 (mean)	KPS, RNLI	No significant factors found.

Publications focusing on more than one tumor identity have been marked with an asterisk (*). These publications were discussed for every single tumor identity.

QoL, as indirectly measured by the KPS, generally shows improvement after surgery, indicating an enhancement in patients’ functional status (121, 122). Comparing different surgical approaches such as the supraorbital keyhole approach, the endoscopic endonasal approach and the unilateral subfrontal approach revealed no significant differences in QoL outcomes. Furthermore, the choice of surgical approach does not significantly impact the rates of gross total resection or postoperative vision outcomes, suggesting no indirect influence on QoL through these factors (122, 123).

### Vestibular schwannomas

Given the close proximity of vestibular schwannomas to critical structures such as the facial and vestibulocochlear nerves, surgical resection of these tumors can result in significant neurological deficits such as facial palsy, hearing loss or vertigo (124, 125). The results of our findings are summarized in **Table 11**.

**Table 11:** Studies investigating QoL in patients after vestibular schwannoma surgery.

First Author	Time of Surgery	Patients (n)	Surgical Approach	Follow-Up (months)	QoL Instruments	Factors Influencing QoL
(126)	2016-2017	7	Endoscopic transcranial transpromontorial	12,9 (median)	SF-36	No significant factor.
(127)	1981-1992	257	Not specified	51,6 (median)	Modified EORTC questionnaire	Improved postoperative QoL is associated with tumors smaller than 1.5 cm in size.
(128)	Not specified	53	Not specified	363	Modified GBI	Older patients experienced improved QoL.
(129)	Not specified	90	Translabyrinthine or retrosigmoid approach	> 18	SF-36	No significant factors; decreased postoperative QoL in 7/8 SF-36 items.
(130)	Not specified	1657	Translabyrinthine or retrosigmoid approach	96 (mean)	Custom Questionnaire	Young age, female sex, and retrosigmoid approach linked to increased postoperative headache.
(131)	1997-2001	42	Middle Cranial Fossa Approach	37 (median)	SF-36	No significant factors; decreased postoperative QoL in 8/8 SF-36 items.
(132)	2001-2003	33	Not specified	< 6	SF-36	No significant factors; postsurgical SF-36 scores normalized within 3 months.
(133)	1999-2007	121	Translabyrinthine or retrosigmoid approach	> 6	SF-36	No significant factors; postsurgical QoL nearly equivalent to healthy population.
(134)	2017-2020	63	Middle Cranial Fossa Approach	7 (mean)	WRS, PANQOL	Hearing preservation associated with higher QoL.
(124)	2005-2011	117	Middle Cranial Fossa Approach	> 6	SF-36	Postsurgical vertigo and impaired hearing status negatively impact QoL.
(125)	Not specified	398	Not specified	12 (median)	FaCE Scale	Facial palsy reduced QoL, particularly affecting social life in younger patients.
(135)	Not specified	397	Not specified	> 120	PANQOL	No difference in short-term (<6 years) or long-term (>10 years) QoL outcomes between radiotherapy, microsurgery, or combined therapies.
(136)	1996-1999	54/70	Not specified	38,4 (median)	SF-36	Surgical excision significantly reduced social functioning and role limitations due to physical functioning.

Additionally, psychological factors such as depression, anxiety and sleep disorders further compound the challenges, negatively impacting the postoperative QoL in these patients (131).

Contrasting perspectives emerge regarding the overall post-surgical QoL in these patients. Some research suggests that quality of life remains stable postoperatively (126, 133). However, other studies (128, 131, 132, 136) indicate a post-surgical decline in QoL, which appears to normalize within six months post-surgery (132).

Smaller vestibular schwannomas with less than 1.5 cm in diameter have been associated with a more favorable postoperative quality of life (127). This finding is in contrast to other studies (128, 129) who report no significant impact of tumor size on postoperative QoL.

A particularly challenging complication is postoperative facial palsy, which significantly lowers QoL in social domains, notably among younger women under 40 years (125). Hearing preservation has been found critical for postoperative QoL with better preoperative hearing levels correlating with improved postoperative outcomes and QoL (124, 134).

Another aspect is the choice of surgical approach. Postoperative headaches have been linked to the retrosigmoid approach, showing a noticeable decrease in QoL, particularly among younger women, compared to the translabyrinthine or middle cranial fossa approaches (130). Otherwise, it was found that the surgical approach or even the treatment modality (Microsurgery, radiotherapy or combined therapy) generally does not affect postoperative QoL (129).

The economic impact on younger patients is also significant, with some studies noting a decrease in QoL due to financial stress, a factor less impactful on older patients who may possess greater financial reserves or be at a different career stage (128). However, such findings were not consistently reported across all studies (129).

### Clival chordomas

Clival chordomas, although histologically classified as low-grade tumors, demonstrate clinically malignant behaviors due to their diffusely infiltrative growth patterns and high rates of recurrence and tumor-related mortality (137, 138). Given the aggressive nature of the disease and the necessity for comprehensive removal, the challenge of achieving a surgical outcome that effectively manages the disease while also preserving the patient’s quality of life is crucial. The results of our findings are summarized in **Table 12**. The endoscopic endonasal approach has become a popular approach for resecting clival chordomas as it offers reduced morbidity compared to more extensive transcranial and transfacial approaches (141, 142).

**Table 12:** Studies investigating QoL in patients after chordoma surgery.

First Author	Time of Surgery	Patients (n)	Surgical Approach	Follow-Up (months)	QoL Instruments	Factors Influencing QoL
(139)	2002-2010	40	Microsurgery vs. Gamma knife	Up to 60	KPS	No difference in KPS scores between groups at follow-up.
(86)*	2010-2011	6/66	Endoscopic endonasal	Up to 6	ASBQ, SNOT-22	Improved short-term QoL with gross total resection.
(84)*	2008-2011	8/85	Endoscopic endonasal	Up to 6	ASBQ, SNOT-22	Improved short-term QoL with gross total resection.
(71)	Not specified	38	Endoscopic endonasal	Up to 3	SF-36, RSOM-31	Vascularized flap reconstruction further decreased postoperative QoL.
(93)	Not specified	88	Not specified	Not specified	SF-36, KPS, PH-Q9	Neurological deficits, pain medication use, corticosteroid treatment, and depression levels impact QoL.
(81)*	2009-2012	3/55	Endoscopic endonasal transclival	Up to 12	ASBQ	No negative long-term QoL impact from skull base surgery via expanded endonasal approach.
(140)	1999-2018	167	Mainly endoscopic endonasal transclival	Up to 264	Katz-Index	No factors influencing postsurgical QoL.

Publications focusing on more than one tumor identity have been marked with an asterisk (*). These publications were discussed for every single tumor identity.

Studies indicate that even extended endoscopic endonasal approaches do not negatively influence long-term QoL and only lead to temporary short-term impairments in general and sinonasal QoL (84, 86, 139). Comparisons with other treatment modalities, such as gamma knife surgery, also show no difference in QoL (139).

Gross total resection significantly improves the recovery of postoperative sinonasal QoL (84, 86). The use of a vascularized flap in endoscopic endonasal surgery is associated with more pronounced sinonasal symptoms compared to approaches that do not utilize the flap. Specifically, studies have indicated that such approaches can negatively affect physical and mental QoL at least up to three months post-surgery (71), highlighting the need for careful consideration of surgical techniques to minimize these effects. Additionally, the use of corticosteroids and pain medication correlates with reduced QoL after surgery (93).

Most studies utilize sinonasal QoL instruments. However, it should be noted that the resection of clival chordomas can lead to a variety of complications, such as neurological deficits or CSF leaks, which can increase the burden of the disease for the patient. Neurological deficits such as sensory deficits and bowel and bladder dysfunction can significantly impact the QoL in these patients and diplopia has been linked to anxiety and depression and was often already present prior to surgery (93). While gross total resection should be attempted, avoiding neurological deficits is paramount to preserving the patient’s QoL.

## Discussion

This systematic review represents the first comprehensive evaluation of factors that influence QoL following the resection of skull base tumors across various anatomical locations. Whereas previous reviews have primarily focused on specific areas, such as the anterior skull base (143), or on particular approaches like the endoscopic endonasal approach (144), our extensive review covers a broad range of skull base locations and surgical techniques. This approach provides a more holistic perspective on postoperative QoL in patients with skull base tumors.

However, this literature review also demonstrates that most publications dealing with quality of life focus on the anterior skull base and the endoscopic endonasal approach. Hence, the most common used tools in this review were the SNOT -22 and the ASBQ, mainly evaluating the sinonasal outcome and quality of life. This leads to a potential bias, as other aspects of quality of life or other surgical approaches are less frequently discussed.

Our examination of the literature has revealed several key factors that may impact QoL following surgery.

### Sociodemographic factors

We identified age and gender as two key sociodemographic factors that influence QoL after surgery.

Research has consistently shown that female gender is associated with poorer QoL outcomes in various skull base tumors (24–26, 37, 43, 96, 98, 100). The mechanism for this disparity is not clear and may stem from a combination of biological, psychological and social factors. Biologically, hormonal differences could influence symptom severity and recovery trajectories (145). Psychologically, women may experience higher levels of distress or depression related to diagnosis and treatment, which can adversely affect QoL (146, 147). Socially, women often face greater challenges in balancing treatment with familial and caregiving responsibilities (148). This complex interplay highlights the need for gender-specific considerations in the management and support structures for tumor patients to optimize their QoL after surgery.

Age also appears to be a significant determinant of QoL. Numerous studies have demonstrated that older patients often experience a reduced QoL following the resection of skull base tumors (45, 98, 100). Conversely, research indicates that younger patients may suffer a more rapid deterioration in QoL compared to older individuals. This may be attributed to the greater economic impact experienced by younger patients, who often face substantial challenges in balancing recovery with employment and financial responsibilities (49, 128).

### Tumor localization

Patients undergoing surgery for meningiomas in the anterior or middle cranial fossa generally report a higher postoperative QoL compared to those with tumors located in the posterior fossa (13). The proximity of posterior fossa tumors to critical brainstem and neurovascular structures means that more aggressive resections in this area tend to lead to neurological deficits, which are strongly correlated with reduced quality of life QoL for patients (103). However, in cases of petroclival meningiomas where the brainstem was compressed preoperatively, patients generally experience a significantly improved QoL after surgery (107).

Regardless of the tumor entity, QoL in patients with anterior skull base tumors typically declines immediately following resection. However, it generally returns to baseline levels within 6 to 12 months postoperatively (24, 28, 37). Endonasal approaches may initially disrupt nasal and sinus function, resulting in temporary discomfort and a reduced QoL, particularly in the sinonasal domain.

### Tumor entity

Individuals with malignant pathologies, particularly in the anterior skull base, exhibited significantly lower QoL scores six months after surgery compared to patients with benign lesions. However, these patients demonstrated considerable improvements in QoL twelve months after surgery. In contrast, patients with benign tumors tended to experience a more stable QoL throughout their postoperative recovery period (24, 31).

The majority of studies examining meningioma resections at various skull base locations have shown a significant improvement in QoL after surgery (98, 100, 121, 122). Conversely, a smaller number of studies report no change in QoL following the surgical intervention (13, 99). Upon closer examination of meningioma location, petroclival meningiomas and medial sphenoid wing meningiomas are notably associated with a negative impact on QoL. This correlation might be attributed to poor neurological functional outcomes and the highest recurrence rates among meningiomas (110–112).

Patients undergoing resection of pituitary adenomas typically experience an improvement in QoL after surgery, following a transient decline primarily due to sinonasal symptoms related to the endonasal approach (43, 74, 82). These patients usually exhibit a good preoperative QoL, and the psychological relief experienced after surgery plays a crucial role in their overall QoL improvement (46). In contrast to tumor size (92), endocrinopathy negatively impacts the QoL for patients with pituitary adenomas (54, 91) and relief from these endocrine disorders has been linked to improved QoL outcomes (43). Patients with prolactinomas may experience improvements in QoL as early as three months after surgery (43), whereas those with acromegaly or Cushing disease generally require significantly more time to recover their QoL (43, 149). This difference may be attributed to the residual effects on appearance, mood and metabolism that persist even after hormonal levels have normalized (150–152) However, it is important to note that examining QoL specifically related to endocrinopathy falls beyond the scope of this review and has been extensively discussed in previous reviews (153, 154).

### Surgical approach

For most skull base tumors, a variety of surgical approaches are utilized for tumor resection. The choice of approach generally depends on the surgeon’s experience and preference.

However, particularly for tumors located in the pituitary region and the anterior skull base, endoscopic approaches have been widely adopted due to their minimally invasive nature and the panoramic view they provide the surgeon. While endoscopic endonasal approaches are associated with a higher incidence of CSF leaks (24, 26, 73, 75, 121, 123), our findings indicate no significant impact on the QoL for patients from these leaks. However, the prophylactic insertion of a lumbar drain has been associated with poorer QoL after surgery, persisting as long as 12 months after the procedure. Patients who received lumbar drains experienced higher morbidity, longer hospital stays and a reduction in QoL potentially stemming from associated side effects such as discomfort, headaches or infections (34). In contrast, the use of nasoseptal flaps for reconstruction and prevention of CSF leaks is correlated with worsened rhinologic symptoms and headaches in the immediate postoperative period. However, these effects do not appear to impact long-term QoL (62, 66, 71, 80, 86, 91).

Few studies have compared different surgical approaches and their impact on QoL. Such comparisons were primarily limited to variations of the endonasal approach, which revealed only minor differences in long-term sinonasal QoL, particularly with expanded endoscopic approaches used for more complex tumors (50, 51, 61, 78, 79). However, most studies we have included lack comparisons of different open transcranial approaches or the comparison between open and endonasal approaches in terms of perceived QoL outcomes for patients.

### Gross total resection and neurological deficits

Gross total resection (GTR) is the objective in most tumor surgeries, whenever feasible. This is particularly crucial in malignant tumors, where achieving complete resection is associated with longer survival and reduced recurrence rates. However, achieving GTR in skull base tumors often presents numerous challenges due to the proximity to critical neurovascular structures.

The studies included in this review indicate that the quality of life following GTR of skull base lesions generally improves or remains unchanged, irrespective of the surgical approach employed. The positive effect is particularly evident in cases of craniopharyngioma, where GTR is often linked to a significantly enhanced QoL. The correlation is likely due to the reduced likelihood of tumor recurrence, the decreased need for subsequent surgical interventions and the reduced necessity for adjuvant radiotherapy (96). Although pursuing GTR in cases of craniopharyngiomas may result in endocrinopathy, the overall benefits of GTR seem to outweigh the decrease in QoL caused by new endocrine disorders (96, 155).

In contrast, patients with petroclival meningiomas often experience a deterioration in QoL after gross total resection (105, 107). This decline may be attributed to the vastly different spectrum of complications associated with resecting petroclival meningiomas compared to craniopharyngiomas. The proximity of petroclival meningiomas to the lower cranial nerves and the brainstem significantly increases the likelihood of neurological deficits, which are associated with poor postoperative QoL (107). Therefore, it is necessary for the surgeon to balance the pursuit of gross total resection with the patient’s QoL after surgery and tailor the surgical plan for each individual patient (109).

In meningioma patients, a more aggressive resection tend to lead to a greater incidence of cranial nerve deficits, which can significantly hinder a patient’s ability to return to normal life and substantially diminish their QoL (44, 103). However, not all cranial nerve deficits uniformly impact QoL in the same way.

The severity and type of deficit play critical roles in determining the extent of impact. For example, cranial nerve deficits affecting motor function and thus enabling actions such as swallowing, may be more debilitating and disruptive compared to sensory deficits. Particularly, changes in vision significantly influence QoL both before and after surgery, with postoperative improvements in vision strongly correlating with enhanced QoL for the patient (23, 39, 57, 100, 156). While some publications consider the loss of olfaction or taste to be less impactful (45), the patient’s occupation and leisure activities can significantly influence how anosmia affects their quality of life (157).

Furthermore, the individual’s ability to adapt to these changes also varies, with some patients managing to find effective coping strategies that mitigate the impact on their daily lives. This complexity underscores the need for a personalized approach in postoperative care, aimed at addressing specific deficits and supporting overall well-being.

Vestibular schwannomas present significant challenges that can impact postoperative quality of life, with outcomes varying widely across different studies and neurosurgical centers. Due to the proximity to the facial and vestibulocochlear cranial nerves, complications typically result in neurological deficits related to their functions. Notably, younger women may experience drastic impairments in QoL due to postoperative facial palsy (125), whereas hearing loss affects QoL independently of gender (124, 134). Although the size of the tumor significantly influences the complexity of the surgery, its impact on QoL is less clear. Only one study has found a correlation between larger tumor size (> 1,5cm) and worse postoperative QoL (127), whereas two other studies reported no impact on QoL (128, 129).

### Implications for clinical practice

The presented literature offers several key insights for clinicians. The evidence consistently shows a transient decline in QoL after surgery across almost all studies, regardless of the tumor’s anatomical location or entity. Interestingly, this decline tends to recover to baseline levels postoperatively and in some cases, particularly with tumors treated at the anterior skull base, patient’s quality of life surpasses preoperative levels. This could be attributed to the predominance of less invasive endoscopic surgeries in this region, which are associated with faster recoveries and less impactful long-term sinonasal outcomes compared to traditional open surgeries (158). However, we found no clear evidence demonstrating that endonasal approaches are superior to open approaches with regard to quality of life.

It is important to highlight that changes in QoL are significantly influenced by the patient’s preoperative clinical status. Patients who were asymptomatic prior to surgery often experience a deterioration in QoL postoperatively (37). This observation brings to light the complexity of measuring QoL of patients who undergo surgery not because of current symptoms but to prevent future complications, a common scenario in skull base tumors. This preventative aspect of surgical intervention is often not captured in QoL assessments, emphasizing the need for developing more nuanced survey instruments that can capture the preventative necessity of skull base surgery.

However, our review of the current literature highlights the significant impact of non-modifiable factors such as age and sex on QoL outcomes, alongside modifiable factors like psychological support. Early psychological interventions, especially for patients undergoing treatment for complex tumors, appear to enhance QoL, suggesting the importance of integrated care models that address both physical and mental health after surgery (41).

Moreover, the severity of the tumor (malignant versus benign), the necessity of radiotherapy and recurrent surgeries are predictors of poorer QoL outcomes (31, 37, 96). This underscores the need for a tailored follow-up strategy that allocates more resources to high-risk patients to mitigate these effects.

Gross total resection, while often the primary goal in skull base surgery, should not always be considered, if followed by cranial nerve or other neurological deficits, diminishing the quality of life of patients. Surgical planning should include the patient’s individual perception which neurological deficits they could endure. This often depends on the patient’s occupation or leisure activities, making this decision highly individual.

The demographic characteristics of the skull base tumor population present additional challenges. Many patients are elderly with multiple comorbidities and depending on the tumor and treatment type, may have a shortened life expectancy. These factors complicate data collection and longitudinal study follow-ups, making large-scale, statistically significant conclusions difficult. Moreover, the histological variability of these tumors adds another layer of complexity in interpreting the impact on QoL.

It is crucial to recognize the multifaceted nature of QoL and the potential discrepancy between patient-reported outcomes and clinical assessments by healthcare providers (102). Regular collection of self-reported QoL data is vital, particularly given the improving survival rates for patients with skull base tumors. Such data not only provide insights into the patient’s recovery trajectory but also help in adjusting care plans to enhance overall well-being of the patients.

## Limitations

Our study has several limitations. This literature review was conducted using PubMed, other databases were not explored. Consequently, some studies addressing quality of life following skull base tumor resection may have been omitted. However, additional targeted literature searches were performed to address underrepresented tumor types. To our knowledge, this is the first review encompassing many different tumor types and anatomical localizations.

The variability in scores, tumor types, localizations and treatment modalities across the studies presented prevented direct comparisons. Therefore, this review cannot provide definitive conclusions regarding quality of life. Nevertheless, it offers insights into potential influential factors.

Most studies included in this review focus on anterior skull base tumors and the endoscopic endonasal approach. Consequently, the most frequently used assessment tools were the SNOT-22 and the ASBQ, which predominantly evaluate sinonasal quality of life. This focus may introduce bias, as other aspects of quality of life and different surgical approaches are less frequently discussed.

Additionally, this review only considered publications related to surgical treatment of skull base tumors and did not explicitly evaluate the impact of radiotherapy, conservative treatments, or other treatment modalities.

## Conclusion

The transient decrease in QoL following skull base tumor resection is a commonly observed outcome across various anatomical locations and tumor entities. The recovery timelines and outcomes are influenced by a wide variety of factors such as tumor entity, anatomical localization, surgical techniques, patient demographics, and psychosocial considerations. Recognizing and addressing the factors influencing QoL is important for improving patient outcomes and emphasizing individualized care.

## Data Availability

The original contributions presented in the study are included in the article/supplementary material Further inquiries can be directed to the corresponding author/s.
